# Occupational Exposure to Blood and Body Fluids among Health Care Workers in Gondar Town, Northwest Ethiopia: A Result from Cross-Sectional Study

**DOI:** 10.1155/2020/3640247

**Published:** 2020-05-15

**Authors:** Giziew Abere, Dawit Getachew Yenealem, Sintayehu Daba Wami

**Affiliations:** Department of Environmental and Occupational Health and Safety, Institute of Public Health, University of Gondar, P.O. Box 196, Gondar, Ethiopia

## Abstract

**Background:**

Health care workers are at the greatest risk of developing blood-borne diseases through occupational exposure to blood and other contaminated body fluids. Occupational exposure to blood and body fluids (BBFs) continues to be the major public health problems and serious concern for the health care force in Ethiopia. Therefore, this study was aimed to determine the prevalence of exposure to blood and other body fluids and its associated risk factors among health care workers.

**Methods:**

The institution-based cross-sectional study design was employed from January 20 to February 30, 2018. A stratified random sampling followed by a simple random sampling technique was used to select 286 study participants. Data were collected using a pretested and structured questionnaire. Bivariate and multivariable logistic regression analyses were performed to identify factors associated with occupational exposure to BBFs. The significance level was obtained at a 95% confidence interval (CI) and *p* value ≤ 0.05.

**Results:**

The prevalence of occupational exposure to blood and body fluids among health care workers in the last 12 months was 65.3% (95% CI: 59.4, 70.9). Lack of readily available personal protective equipment (adjusted odds ratio (AOR)) = 3.01, 95% CI: 1.56, 5.84), lack of training (AOR = 3.36, 95% CI: 1.1, 11.2), Khat chewing (AOR = 2.74, 95% CI: 1.3, 5.8), and being a medical doctor (AOR = 5.1, 95% CI: 1.68, 15.21) were significantly associated risk factors with occupational exposure to blood and other body fluids.

**Conclusions:**

In this study, occupational exposure to blood and other body fluids among health care workers remains a major health problem. Hence, ensuring the availability of personal protective equipment, developing strategies on banning, and strict monitoring of Khat chewing and training on infection prevention should be emphasized to minimize the problem.

## 1. Introduction

Worldwide occupational exposure to blood and body fluids is a major health care-related problem [1] that becomes ubiquitous means of exposure to blood-borne pathogens [2]. According to different studies from 35 million health care workers (HCWs), 2 [3] up to 3 [1, 4] millions are exposed to blood-borne diseases. As a consequence of occupational exposures, 66,000 HBV, 16,000 HCV, and 1,000 HIV infections occur among HCWs each year [5, 6].

Occupational exposure to blood and body fluids is accidental contact with blood and body fluids during a medical intervention by HCWs [7]. It can result from percutaneous and mucocutaneous injury or blood contact with nonintact skin [8]. The most common means of exposure to blood and body fluids in health care sectors happen to be needle stick and sharp injuries [9–12] and a considerable number of researches equate both [13]. In light of this evidence, worldwide estimates indicate that 1 in 10 health care workers experience a sharp injury every year [14, 15].

The risk of accidental exposure to blood and body fluids is linked to activities like taking blood samples [16, 17], giving injections [16, 18], recapping of already used needles [13, 16, 18–20], surgery [16, 21], delivery, giving emergency care, cleaning up transportation of waste products [16, 19], and instrument processing procedures [13, 16, 18, 19]. While engaging in such routine clinical activities in health facilities, HCWs are at risk of occupationally acquired infections via blood and body fluid exposure [9, 22, 23].

Although CDC devised standard precautions as the best intervention to prevent blood and body fluid exposures [24], it has been shredded with inadequate and nonuniform adherence across practitioners [25] which can be attributed to underestimation of the hazard [10]. Because many people with blood-borne infections do not have symptoms, it is necessary to apply standard precaution measures to all clients and patients [26]. Unfortunately, despite the simplicity and clarity of these precautions, the practice among HCWs in the clinical settings is low, especially in resource-limited settings [27], thus exposing HCWs to the risk of infection [25, 28]. Furthermore, the World Health Organization (WHO) has recommended governments to transition to the exclusive use of safety injection devices by 2020 [29].

Regardless of the efforts taken, findings indicate that health care workers are at high risk of contracting blood-borne illnesses such as HCV, HBV, and HIV [15] that is associated with significant mortality and morbidity of health care workers [30].

According to the study in 21 African countries, the estimated pooled 12-month prevalence of occupational exposure to body fluids was found to be 48.0% [2]. In another study, the prevalence of occupational exposure to BBF is 79% [31]. The magnitude of blood and body fluid exposures in Ethiopia ranges from 29.2% to 65.9% [4, 22, 32].

The occupational risk of exposure to BBFs and needle stick injuries not only affects the safety and wellbeing of HCWs [33] but also compromises the quality of health care delivered [1, 4], and a decrease of the attrition rate also indicated consequences that eventually lead to a shortage and crisis [34, 35]. Reports showed that occupational risks are also the main triggering issues in strikes and other complaints by health force in low-income countries [36]. Moreover, occupational blood exposure results in substantial psychological problems, such as job-related depression and anxiety [37] and considerable management costs ranging from 650 to 750 US dollars [38]. Even the fear of occupational transmission of BBV may cause HCW to change occupations [39].

Based on the evidence generated from different research outputs, there is an association between work experience [4, 19, 32], type of profession [32, 40], workplace factors like lack of training on IP [17, 32, 40], and occupational exposure to blood and other body fluids. In addition, inconsistent use of PPE particularly gloves and not complying with standard precautions [4, 32], sex [17, 19], being single [17], and older age are implicated as associated factors [19].

Even though efforts have been taken, occupational exposure to blood and body fluids continues to be the major public health problem and is a serious concern for the health care force in Ethiopia. Occupational exposure to blood and body fluids demands updated information regarding its magnitude and associated factors since there is dynamism in health care force composition and behaviors. Such a study will have a significant input in the formulation and revision of the appropriate strategy to modify and facilitate the overall prevention of occupational exposure to BBFs. Therefore, this study aimed to determine the current prevalence and associated factors in Gondar town health care facilities.

## 2. Materials and Methods

### 2.1. Study Design and Setting

The institutional-based cross-sectional study design was employed. The study was conducted in Gondar town from January 20 to February 30, 2018. Gondar town is located north of Addis Ababa at 737 km and was founded in 1635. Based on the 2017 national census conducted by the Central Statistical Agency of Ethiopia (CSA), Gondar had a total population of 360,600. In Gondar, there are eight health centers and one referral hospital, which had a total of more than 1004 health care workers.

### 2.2. Study Population

Health care workers who were working at hospitals and health centers in Gondar town with one-year experience were included in the study. However, health care workers who were in annual leave and who were critically ill during the data collection period were excluded from the study.

### 2.3. Sample Size and Sampling Technique

The sample size was determined using single population proportion formula assuming a population proportion of 65% [4], a margin of error 5%, and confidence interval of 95%; since the total population was less than 10,000, we have used reduction formula resulting in 286 health care workers.

A stratified random sampling technique followed by simple random sampling was used to select 286 study participants using a proportional allocation from the nine health institutions by considering each health center and hospital as a stratum.

### 2.4. Operational Definition

Health care workers, for this study, are defined as workers essential to provide health care delivery and have direct and indirect contact with patients. Those include health officers, nurses, midwives, medical doctors, pharmacists, and medical laboratory professionals.

Occupational exposure to blood and body fluid refers to reasonably anticipated skin, eye, mucous membrane, or parenteral contact with blood, body fluids, or other potentially infectious material that may result from the performance of one's professional duties [41].

Body fluids are liquids originating from inside the bodies of living people. They include fluids that are excreted or secreted from the body.

The availability of PPE is the accessibility of specialized clothing or equipment worn by professionals for protection against health and safety hazards in the health facilities.

Khat chewer is a health care professional chewing Khat (a mildly psychoactive substance) used three times a week for at least 1 year [42].

### 2.5. Data Collection Tools and Procedures

Data was collected using a pretested and structured questionnaire through a self-administered technique. The questionnaire was developed from different studies in the literature to assess the health care worker's exposure to BBFs and had three parts. The first part covered sociodemographic characteristics, the second part covered the behavioral and working environment variables, and the third part covered the occupational exposure of HCWs to blood and other body fluids.

### 2.6. Data Quality Control

The training was given for data collectors and supervisors for two days on procedures, techniques, and ways of data collection. Before the commencement of the actual data collection process, the questionnaire was pretested at Debre Tabor District Hospital. Besides, continuous and strict supervision was carried out during the data collection process.

### 2.7. Data Processing and Analysis

Data was entered using Epi-Info version 7.1 and cleaned and analyzed using SPSS (Statistical Package for Social Sciences), version 20.0. Descriptive statistics such as frequency, mean, percentage, and standard deviations were calculated to describe the characteristics of the study population in relation to different variables. The binary logistic regression model was fitted to identify factors associated with BBFs. Blood and other body fluids were regressed against the sociodemographic, behavioral, and work environment factors separately. Before fitting the binary logistic regression model, the goodness of model fit test was checked by Hosmer and Lemeshow test, and the assumption was satisfied (*p* value > 0.05). The significance level was obtained at 95% CI and *p* value ≤0.05. The adjusted odds ratio was used to determine the strength of association.

## 3. Results

### 3.1. Sociodemographic Characteristics of Respondents

A total of 277 HCWs participated in the study, which gives a response rate of 96.8%. The majority, 165 (59.6%), of health professionals in this study were males, and the age of 198 (71.4%) of the respondents was in the range of 25–32 years. Regarding their educational level, the majority, 184 (66.4%), of the study participants had a B.S. degree, and 84 (30.3%) were nurses. Below a quarter, 60 (21.7%) were working in outpatient departments, and 104 (37.5%) had 3–5 years of work experience (Table 1).

### 3.2. Behavioral Characteristics of Respondents

The majority, 264 (95.3%), of the study participants had used at least one personal protective equipment (PPE) during the last health care procedure. Above half, 171 (61.7%), of health care workers washed their hands before and after any health care procedure or handling of BBF-contaminated waste, and only 135 (48.7%) of the study participants complied with the standard precautions.

Of the total study participants (277), 74 (26.7%) of the respondents were Khat chewers and 206 (74.3%) of the respondents drank alcohol; among them, a high proportion, 133 (64.5%), drank occasionally. Ninety-six (34.7%) of health care workers reported that they had a sleeping problem (Table 2).

### 3.3. Work Environment Factors

The majority, 253 (91.3%), of the respondents had taken training on infection prevention. More than half of the study participants (53.1%) reported that there was readily available personal protection equipment (PPE) available over the past year. Nearly half, 133 (48%), of the study participants reported the presence of safety signs in their working unit. Only 95 (34.3%) of the study participants reported that there were enough hand washing basins in their department of work. Though 155 (56%) of the study participants reported that there was no infection prevention committee in the health care facility, the same number of study participants reported that there was workplace safety service for the prevention of occupational exposure to BBFs (Table 3).

### 3.4. Prevalence of Occupational Exposure to Blood and Body Fluids

Of the total, 241 (87%) study participants had been exposed to blood and other body fluids in their lifetime. Moreover, the lifetime prevalence of the sharp injury of health care workers was 73.3% (95% CI: 67.7, 78.4). However, 181 (65.3%) (95% CI: 59.4, 70.9) of health care workers were exposed to BBFs in the past year. Among those exposed, almost half, 90 (49.7%), of them were exposed two or three times per year (Table 4).

### 3.5. Factors Associated with Occupational Exposure to Blood and Body Fluids

The multivariable logistic regression analysis showed that the lack of readily available/shortage of personal protective equipment, lack of training on infection prevention, Khat chewing, and profession/being a medical doctor were found to be significantly associated risk factors with occupational exposure to blood and other body fluids at *p* value <0.05 (Table 5).

In this study, HCWs who perceived a lack of readily availability PPE in the health facility had a higher risk of BBF exposure. Health care workers who reported that there is lack of readily available/shortage of PPE had 3.01 times higher odds of exposure to blood and body fluids when compared to those who had perceived that there was readily accessible personal protective equipment (AOR = 3.01, 95% CI: 1.56, 5.84).

Lack of training on infection prevention was associated with a high likelihood of experiencing BBF exposure. Health care workers who did not take training on infection prevention had 3.36 times higher odds of being exposed to BBF compared to those who had taken training (AOR = 3.36, 95% CI: 1.1, 11.2). Health care workers who chew Khat had 2.74 times higher chance of being exposed to blood and body fluids compared to non-Khat chewers (AOR = 2.74, 95% CI: 1.3, 5.8). In addition, study participants who are medical doctors in the profession had 5.1 times higher odds of exposure to BBF when compared to nurses (AOR = 5.1, 95% CI: 1.68, 15.21).

## 4. Discussion

In this study, the period prevalence of BBF among HCWs was high. Being a medical doctor, lack of training, Khat chewing, and readily unavailability of PPE were the significant predictors for the BBF exposure.

According to this study, the prevalence of exposure to blood and other body fluids among health care workers during the last 12 months was 65.3% (95% CI: 59.4, 70.9). This result showed that a high proportion of health care providers are exposed to blood and other body fluids which indicated that they are threatened by the transmission of blood-borne pathogens including Hepatitis B Virus (HBV), Hepatitis C Virus (HCV), and HIV/AIDS virus. The finding is in agreement with the prevalence rate reported in Bahir Dar (65.9%), Mizan Tepi (64.1%), and Kaduna state in Nigeria (68%) [1, 4, 19]. However, the prevalence rate in our study was higher than the study done in Addis Ababa (41.3%), Johannesburg metropolitan district (25.2%), Tanzania (48.6), Lebanon (30%), Fako division (50.9%), Tehran (53.4), and Kenya (25%) [32, 41, 43–47]. This difference may be attributed to the difference in regular training about safety precautions and infection prevention, inadequate supervision by health administrators, and infrastructure development [7]. In addition, poor workplace safety and a high load of patients in the facilities in our study setting could not be ruled out.

In this study, physicians were at high risk of exposure to blood and body fluids. Though some studies have shown that nurses have higher blood and body fluid exposure rates than physicians [48, 49], there have been some studies reporting higher rates among physicians [1, 50–52]. The latter is further supported by this study. This might be explained due to the fact that physicians are few in numbers and they work in different clinical procedures and relatively spent long working hours. This, in turn, makes them exhausted and put them at high risk of exposure to blood and body fluids.

Lack of readily available/shortage of personal protective equipment was significantly associated with occupational exposure to blood and body fluids. This is supported by the study done in Ethiopia [32, 53] and China [54] in which a shortage of available personal protective equipment was indicated as a factor associated with occupational exposure to blood and other body fluids. This finding might explain that the ready availability of personal protective equipment enabled health care workers to use personal protective equipment that has the potential to prevent the exposure of contaminated blood and body fluids. This, in turn, gives a hand to comply with standard precautions. Besides, frequent inaccessibility of personal protective equipment could decrease the motivation of previously energetic health care providers and could be a reason to be exposed to contaminated blood and body fluids. Furthermore, the unavailability of PPE might have a psychological effect on HWs. It can distract their concentration; they become dissatisfied and finally might lead them to stress, which will, in turn, be a risk for BBF exposure.

Moreover, the lack of training on infection prevention was a risk factor for occupational exposure to blood and body fluids. Health care professionals who did not take training on occupational infection prevention had higher odds of occupational exposure to BBF than those who had taken the training. This result is in agreement with the study findings from regional hospitals in Ethiopia [55], Addis Ababa [32], Jimma [17], and Tanzania [56]. This might be because training on infection prevention increases the chance of getting updated information and skills about procedures and mechanisms which might reduce the likelihood of occupational exposure to blood and body fluids [57]. Moreover, as the training enabled health care workers to know the consequence of blood and body fluid exposure, their adherence to standard precaution tends to be increased; thereby, the chance to be exposed to blood and body fluids would be decreased.

Furthermore, health care workers who chew Khat had reported a higher chance of exposure to blood and other body fluids compared to non-Khat chewers. This might be due to its inherent effect on their behavior and it impaired workers' concentration and performance, anxiety, and trouble sleeping. A high blood level of such substances during work will endanger both safety and efficiency and will be the cause of increased likelihood of mistakes, not adhering to standard operating procedures, poor decision making, and errors in judgment. This is supported by the study done in Ethiopia in which Khat chewing was identified as significantly associated with workplace injury [42, 58]. Moreover, the study done in the UK identified that Khat chewing has been found to decrease work capability and increase the rate of accidents [59].

### 4.1. Limitations of the Study

This study has shared the limitations of cross-sectional studies, the difficulty of determining causal relationships between BBFs and predictor variables. In addition, the possibility of recall bias could not be ruled out since worse and recent BBF exposure was better than the less serious and older ones.

## 5. Conclusion

A higher proportion of health care providers were found to be exposed to BBFs in this study. Lack of readily available/shortage of personal protective equipment, Khat chewing, and lack of training on infection prevention were found to be predictors of occupational exposure to BBFs among health care workers. Therefore, ensuring ready availability of personal protective equipment, developing strategies on banning, strict monitoring and education on Khat chewing, and training on infection prevention should be emphasized to minimize occupational exposures to BBFs.

## Figures and Tables

**Table 1:** Distribution of sociodemographic characteristics of study participants in Gondar town, northwest Ethiopia, 2018 (*n* = 277).

Variable	Frequency	Percent (%)
Sex		
Male	165	59.6
Female	112	40.4

Age		
≤24	33	11.9
25–27	107	38.6
28–32	91	32.9
≥33	46	16.6

Level of education		
Diploma	51	18.4
Degree	184	66.4
Masters and above	42	15.2

Marital status		
Single	165	59.6
Married	112	40.4

Profession		
Nurse	84	30.3
Medical doctor	48	17.3
Laboratory technology	40	14.4
Health officer	24	8.7
Midwife	65	23.5
Pharmacy	16	5.8

Department		
Outpatient	60	21.7
Injection and dressing	27	9.7
Surgical	18	6.5
Pediatrics	42	15.2
Gynecology	42	15.2
Medical	11	4.0
Laboratory	39	14.1
Others	38	13.7

Work experience		
≤2 years	98	35.4
3–5 years	104	37.5
≥6 years	75	27.1

**Table 2:** Distribution of behavioral characteristics of respondents in health institutions in Gondar City, Northwest Ethiopia, 2018 (*n* = 277).

Variable	Frequency	Percent (%)
Use of PPE		
Yes	264	95.3
No	13	4.7

Practice of washing hands before and after any health care procedure or handling of waste		
Yes	171	61.7
No	106	38.3

Compliance with standard precautions		
Yes	135	48.7
No	142	51.3

Khat chewing		
Yes	74	26.7
No	203	73.3

Drinking alcohol		
Everyday	40	14.4
Once a week	33	11.9
Occasionally	133	48.0
Never	71	25.6

Sleeping problem		
Yes	96	34.7
No	181	65.3

**Table 3:** Institutional factors on occupational exposure to blood and body fluids among health care workers in Gondar town, northwest Ethiopia, 2018 (*n* = 277).

Variable	Frequency	Percent (%)
Training on prevention of infection		
Yes	253	91.3
No	24	8.7

Readily availability of personal protective equipment throughout the year		
Yes	147	53.1
No	130	46.9

Presence of safety signs in their working unit		
Yes	133	48
No	144	52

Presence of enough hand washing facilities in the department or ward		
Yes	95	34.3
No	182	65.7

Presence of infection prevention committee in the health care institution		
Yes	122	44.00
No	155	56.00

Presence of workplace safety service		
Yes	155	56.00
No	122	44.00

**Table 4:** Occupational exposure to blood and body fluids among health care professionals in Gondar town, northwest Ethiopia, 2018 (*n* = 277).

Variable	Frequency	Percent (%)
Lifetime occupational exposure to blood and body fluids		
Yes	241	87
No	36	13

Lifetime sharp injury exposure		
Yes	203	73.3
No	74	26.7

Occupational exposure to blood and body fluids in the past year		
Yes	181	65.3
No	96	34.7

Frequency of BBF in the past one year		
No	96	34.7
Once	62	22.4
Two or three	90	32.5
Four and above	29	10.4

**Table 5:** Bivariate and multivariable analysis of factors associated with occupational exposure to blood and body fluids among health care workers in Gondar town, northwest Ethiopia, 2018 (*N* = 286).

Variable	Occupational exposure to blood and body fluids		COR (95%, CI)	AOR (95%, CI)
	Yes	No		
Sex				
Male	107	58	1.00	1.00
Female	74	38	1.06 (0.63, 1.74)	1.08 (0.58, 2.04)

Age				
≤24	18	15	1.00	1.00
25–27	68	39	1.45 (0.65, 3.20)	1.26 (0.49, 3.21)
28–32	62	29	1.78 (0.78, 4.02)	1.15 (0.34, 3.86)
≥33	33	13	2.11 (0.82, 5.40)	1.14 (0.23, 5.58)

Work experience				
≤2	60	38	1.00	1.00
3–5	67	37	1.14 (0.64, 2.03)	1.42 (0.61, 3.23)
≥6	54	21	1.62 (0.85, 3.11)	2.49 (0.63, 9.89)

Profession				
Nurse	50	34	1.00	1.00
Medical doctors	39	9	2.94 (1.26, 6.86)^*∗∗*^	5.1 (1.68, 15.21)^*∗∗*^
Laboratory technologist	26	14	1.26 (0.57, 2.76)	0.41 (0.03, 5.59)
Health officers	14	10	0.95 (0.37, 2.39)	1.55 (0.47, 5.11)
Midwifery	41	24	1.16 (0.59, 2.26)	2.58 (0.85, 7.82)
pharmacy	11	5	1.49 (0.47, 4.69)	1.61 (0.32, 8.20)

Working departments				
Outpatient department	38	22	1.00	1.00
Injection and dressing	20	7	1.65 (0.60, 4.54)	2.60 (0.81, 8.35)
Surgical departments	14	4	2.02 (0.59, 6.92)	0.87 (0.19, 3.83)
Pediatrics	27	15	1.04 (0.46, 2.36)	0.93 (0.29, 2.91)
Gynecology	25	17	0.85 (0.37, 1.91)	0.46 (0.13, 1.61)
Medical departments	7	4	1.01 (0.26, 3.85)	0.65 (0.12, 3.53)
Laboratories	26	13	1.15 (0.49, 2.70)	4.57 (0.32, 35.00)
Others	24	14	0.99 (0.43, 2.30)	1.20 (0.36, 4.00)

Readily availability of PPE				
Yes	82	65	1.00	1.00
No	99	31	2.53 (1.50, 4.25)^*∗∗*^	3.01 (1.56, 5.84)^*∗∗*^

Training on infection prevention				
Yes	162	91	1.00	1.00
No	19	5	2.13 (0.77, 5.90)	3.36 (1.1, 11.2)^*∗*^

Khat chewing				
Yes	58	19	2.35 (1.26, 4.38)^*∗∗*^	2.74 (1.3, 5.8)^*∗*^
No	123	80	1.00	1.00

*Note.* 1 : 00: reference, PPE: personal protection equipment, ^*∗*^*p* value < 0.05, ^*∗∗*^*p* value ≤ 0.005.

## Data Availability

All data generated or analyzed during this study are included in this article. The data that support the findings of this study are also available from the corresponding author upon reasonable request.

## Abbreviations

AOR: Adjusted odds ratio
BBFs: Blood and body fluids
CI: Confidence interval
COR: Crude odds ratio
HCWs: Health care workers
PPE: Personal protective equipment
SPSS: Statistical Package for Social Science
UK: United Kingdom.
